# M2‐polarized macrophages relate the clearance of gastric lanthanum deposition

**DOI:** 10.1002/ccr3.1989

**Published:** 2019-01-24

**Authors:** Takahito Nakamura, Atsunori Tsuchiya, Masayoshi Kobayashi, Makoto Naito, Shuji Terai

**Affiliations:** ^1^ Department of Internal Medicine Murakami General Hospital Murakami Japan; ^2^ Division of Gastroenterology and Hepatology, Graduate School of Medical and Dental Science Niigata University Niigata Japan; ^3^ EPMA Laboratory, Center of Instrumental Analysis Niigata University Niigata Japan; ^4^ Department of Pathology Niigata Medical Center Niigata Japan

**Keywords:** dialysis, lanthanum, M2 macrophage, phagocytosis

## Abstract

Previous reports have suggested that mucosal barrier failure allows lanthanum to stray into the lamina propria, and macrophages play an important role for the clearance. However, there is no report to analyze the polarity of phagocytizing macrophages. We suggested that M2 macrophage potentially played a major role in the clearance of lanthanum.

Lanthanum carbonate (LC) is an oral phosphate binder to prevent hyperphosphatemia for patients undergoing dialysis. Recently, lanthanum‐deposited cases in the stomach have been reported (Table 1).^1^ On endoscopy, such deposits are recognized as minute whitish granules within mucosa (Figure 1A). Lanthanum deposition in patients taking LC is diagnosed based on detection of macrophages containing foreign body in the lamina propria (Figure 1B). Quantitative analysis of elemental composition by electron probe microanalysis confirms both lanthanum and phosphorus (Figure 1C). Macrophages generally can be divided into pro‐inflammatory type M1 and anti‐inflammatory type M2.^2^ However, the macrophage polarization involved in lanthanum clearance in the gastrointestinal tract has not been elucidated. We found that biopsy specimens from the stomach of 62‐year‐old man with diabetes mellitus, 2‐year history of dialysis, 9‐month history of LC administration, and no specific abnormal physical examination revealed massive eosinophilic infiltration positive for CD68 and CD206 (Figure 1D). CD68 is a common and general marker for both M1 and M2 macrophages, while CD206 is a marker to indicate M2 polarization. Therefore, we assume that phagocytizing M2 macrophages play an important role and M2 polarity induction can accelerate the clearance of lanthanum.^2^

**Table 1:** Previously reported cases of lanthanum carbonate deposition

Authors	Year	Number of patients	Age	Co‐existing symptoms	Duration of LC administration (mo)	Dose of LC (mg/d)	Deposition site	Immunohistochemistry
Yasunaga	2015	1	64	Epigastric discomfort	50	1500	Stomach	CD68, S100, Cam 5.2
Makino	2015	1	63	Heartburn and hiccups	41	500‐750	Stomach	CD68
Haratake	2015	6	61‐69	Abdominal discomfort and pain	21‐49	500‐1500	Stomach and duodenum	CD68, S100, Cam 5.2, Ki‐67
Rothenberg	2015	1	64	Low‐grade fever, nausea, diarrhea, and anorexia	NA	NA	Stomach and duodenum	CD68, CAM5.2, CD1a, S100
Tonooka	2015	1	81	Abdominal discomfort	7	750	Stomach	NA
Goto	2016	19	NA	NA	NA	NA	Stomach and colon	NA
Yabuki	2016	3	68‐77	None	3‐36	750‐1500	Stomach and duodenum	CD68
Shitomi	2017	23	34‐82	Nausea, melena, abdominal pain, and discomfort	3‐67	500‐750	Stomach	CD68
Hoda	2017	5	29‐66	Dysphagia, reflux, nausea, epigastric burn, melena, and early satiety	12‐72	3000‐6000	Stomach and duodenum	NA
Murakami	2017	7	50‐79	Difficulty swallowing and appetite loss	5‐45	NA	Stomach	CD68
Iwamuro	2017	2	42, 73	None	25, 69	NA	Stomach and duodenum	CD68
Hattori	2017	16	47‐79	None	4‐65	500‐1500	Stomach and duodenum	CD68
Nishikawa	2018	1	64	Nausea	72	750‐1500	Stomach	NA
Iwamuro	2018	10	42‐76	NA	12‐86	NA	Stomach and duodenum	NA
Eso	2018	1	26	Epigastric discomfort and appetite loss	84	750	Stomach and duodenum	CD68, PU.1

**Figure 1:**
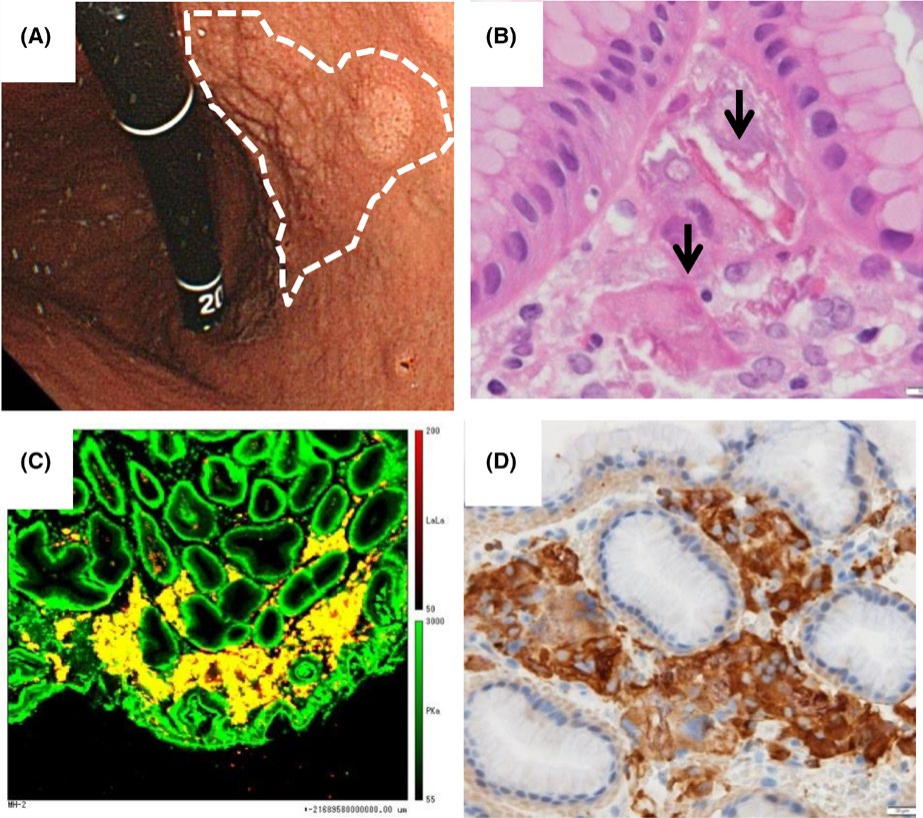
Lanthanum deposition and their analysis (A‐D). Lanthanum deposits are recognized as minute whitish granules within mucosa by endoscopy (A). They are also recognized as eosinophilic infiltration in the lamina propria with hematoxylin and eosin staining (B). Quantitative analysis of elemental composition by electron probe microanalysis reveals macrophages include both lanthanum and phosphorus (yellow area; C). Macrophages are positive for CD206, indicating M2 polarization (D)

## CONFLICT OF INTEREST

None declared.

## AUTHOR CONTRIBUTION

All the authors: made substantial contribution to the preparation of this manuscript and approved the final version for submission. TN and AT: drafted the manuscript. AT: corresponding author. MK and MN: provided clinical support. ST: reviewed the manuscript.
